# Supplementary material for: 3D Imaging and Quantitative Characterization of Mouse Capillary Coronary Network Architecture
Source: Biology (Basel). 2021 Apr 7;10(4):306. doi: 10.3390/biology10040306 (PMC8067837; doi:10.3390/biology10040306)
Supplement: Supplementary file 1 [file biology-10-00306-s001.pdf]

biology

MDPI

Supplementary Materials

3D Imaging and Quantitative Characterization of Mouse Capillary Coronary Network Architecture

Nabil Nicolas and Etienne Roux

Citation: Nicolas, N.; Roux, E. 3D Imaging and Quantitative Characterization of Mouse Capillary Coronary Network Architecture. *Biology* **2021**, *10*, 306. https://doi.org/10.3390/biology10040306

Academic Editor: Pasquale Pagliaro

Received: 01 March 2021
Accepted: 04 April 2021
Published: 7 April 2021

Publisher's Note: MDPI stays neutral with regard to jurisdictional claims in published maps and institutional affiliations.

Copyright: © 2021 by the authors. Licensee MDPI, Basel, Switzerland. This article is an open access article distributed under the terms and conditions of the Creative Commons Attribution (CC BY) license (http://creativecommons.org/licenses/by/4.0/).

Image Processing Commands and Functions

The following document is a step-by-step description of the commands and functions that have been applied for image processing using ImageJ/Fiji software (ImageJ 2.1.0/1.53h/Java 1.8.0_66 (64-bit)).

Downloading *BoneJ* and *3D Suites* plugins is mandatory to fully perform the following instructions.

After each step, the image should be saved as a new TIFF file.

1. Image conversion and calibration

The stack is converted to 8-bits format using the following functions: Image > Type > 8-bit

The stack is calibrated using the following functions: *Image>Properties…>* The voxel width, height and depth are entered.

2. Segmentations

The extraction of the 2 structures of interest is performed by a segmentation technique based on 2 greyscale-defined thresholds. The threshold values are determined from the histogram of frequency distribution of the voxel greyscale values.

The histogram is generated using the following functions: *Analyze>Histogram>* Click *'Yes'* to consider all the images of the stack. The histogram hence appears in a new window.

A threshold is applied using the following functions: *Image>Adjust>Threshold…>* It should be checked that only '*Dark background*' and '*Stack histogram*' are ticked. The minimum threshold value is set using the command *>Apply>* selecting '*Default*' in *Method* and '*Dark*' in *Background*, then ticking '*Black background (of binary masks)*'" *>OK*

3. Filtering

A 3D median filter is applied using the following functions: *Process>Filters>Median 3D…>* Enter '*3*' as a value for x, y and z radii.

4. Skeletonization

A skeletonization is performed using the following functions: *Plugins>Skeleton>Skeletonize (2D/3D)*

5. Distance mapping

The filtered TIFF file is opened.

A distance map is performed using the following functions: *Plugins>3D>3D Distance map>* Enter '*EDT*' in *Map* and check that the filtered image is selectionned > *OK*

The resulted image is a 32-bit type image that should be imperatively converted into a 8-bit type image with the above-discribed method to be able to fuse it with the skeleton

image. (Direct conversion (as in step 1.1), would result in the lost of the information encoded in the image by the distance mapping step). The following steps should hence be attentively performed to avoid wrong results:

a. A conversion factor that will later be used to convert the 32-bit image information into 8-bit information is calculated. For this step, the histogram characteristics of the 32-bit image are obtained using the following command: A*nalyze*>*Histogram*>. The '*X max*' value is noted.

b. The following formula is applied to calculate the conversion factor:

$$Conversion\ factor = \frac{256}{X\ max}$$

c. The conversion factor is applied using the following commands: *Process*>*Math*>*Multiply…* > Enter the conversion factor in *"Value >OK*

d. The brightness and contrast are adjusted using the following functions: *Image*>*Adjust*>*Brightness*/*Contrast…*>*Set*> Enter '*0*' as *"Minimum displayed value"* and '*255*' as *"Maximum displayed value"*>*OK*

e. Finally, the image is converted into 8-bit format using the following command: *Image*>*Type*>*8-bit*

6. Skeleton and distance map fusion

Skeleton and distance map images are fused using the following commands: *Process*>*Image Calculator…* > Select the first and second image and choose the '*AND*' operation. Tick only '*Create new window*' > *OK*

7. Skeleton Analysis

The fused skeleton-distance map image is analyzed using the following commands: *Analyze*>*Skeleton*> *Analyze Skeleton (2D/3D)*. The analysis generates 2 csv tables of data entitled "*Branch information*" and "*Results*".

8. Fractal dimension

The fractal dimension is calculated on the skeleton image using the following functions and commands: *Plugins* > *BoneJ* > *Fractal Dimension* > "Tick '*Automatic parameters*'" > *OK*
